# A Case of Traumatic Hyphema Diagnoses by Telemedicine Between a Remote Island and the Mainland of Tokyo

**DOI:** 10.7759/cureus.65153

**Published:** 2024-07-22

**Authors:** Eisuke Shimizu, Makoto Kamezaki, Hiroki Nishimura, Shintaro Nakayama, Ikuko Toda

**Affiliations:** 1 Ophthalmology, Yokohama Keiai Eye Clinic, Yokohama, JPN; 2 Ophthalmology, Keio University School of Medicine, Tokyo, JPN; 3 Optometry, OUI Inc., Tokyo, JPN; 4 Ophthalmology, Minamiaoyama Eye Clinic, Tokyo, JPN

**Keywords:** d to d, gp, remote medicine, smart eye camera, telemedicine (tm)

## Abstract

Chichijima Island, part of the Ogasawara Islands in Tokyo, is a remote island with a population of approximately 2,000, served by a few resident general practitioners (GPs). This case report discusses the application of teleophthalmology in managing pediatric ocular trauma on this remote island. A pediatric patient sustained an ocular injury from a badminton shuttlecock and was initially examined by a resident GP using a recordable slit-lamp microscope. The ocular images were transmitted to a mainland ophthalmologist through a telemedicine system. The specialist provided remote consultation and recommended further examination and treatment, leading to the patient’s transfer to the mainland. The successful management of this case underscores the critical role of telemedicine in enhancing healthcare delivery in isolated regions. With advancements in medical technology, teleophthalmology is expected to become increasingly vital in providing specialized care in remote and underserved areas. The case highlights the importance of telemedicine in improving access to specialized medical expertise, ensuring timely and effective patient care, and potentially reducing the need for patient transfers to more equipped healthcare facilities.

## Introduction

Telemedicine has revolutionized the delivery of healthcare services, particularly in remote and underserved regions [1]. This case report illustrates the use of teleophthalmology to manage a pediatric patient who sustained an ocular trauma, emphasizing the significance of telemedicine in enhancing healthcare accessibility and outcomes in isolated locations.

Chichijima, situated in Ogasawara Village, Tokyo, is a remote island in the Pacific Ocean, approximately 1,000 km south of mainland Tokyo. It is the largest inhabited island in the Ogasawara Archipelago, with a population of around 2,000 residents. The island is served by the Ogasawara Clinic, which is staffed by three resident physicians, all general practitioners (GP). Access to Chichijima is limited to a weekly ferry service that takes approximately 24 hours each way from Tokyo, necessitating a minimum commitment of five nights and seven days for any trip to and from the island. Despite the presence of basic healthcare infrastructure, specialized medical services are scarce. Ophthalmologists from the mainland visit the island only once a year to conduct specialized outpatient clinics. During these visits, they utilize advanced ophthalmic equipment such as slit-lamp microscopes, fundoscopes, fundus cameras, and fundus three-dimensional optical coherence tomography (OCT) scanners. However, the absence of a resident ophthalmologist creates a substantial gap in the provision of consistent ophthalmological care. Consequently, residents of Chichijima often have to travel to the mainland to receive necessary eye care. This requirement poses significant logistical and financial challenges for the islanders, highlighting the pressing need for improved access to daily ophthalmic services in such isolated communities [2].

Here, we report a case where a GP in Chichijima consulted an ophthalmologist on the mainland for a pediatric patient with eye trauma from a badminton shuttle. Using a telemedicine system, the consultation led to the patient's transfer to the mainland for further examination and treatment. In this case, a teleophthalmology approach was employed, including the use of the recordable slit-lamp microscope, and a telemedicine system, to accurately diagnose and manage the patient’s condition [3-10]​​​​. This case underscores the critical role of telemedicine in bridging the gap between remote locations and specialized medical care, ensuring timely and effective treatment for patients in need.

## Case presentation

A pediatric patient, an eight-year-old Japanese boy, residing on Chichijima Island, sustained an ocular injury caused by a badminton shuttlecock. His medical history included only a left upper arm fracture at age seven, with no prior ophthalmologic diseases or significant family, drug, or allergy histories.

While playing badminton with a friend, the shuttlecock struck his right eye. Immediately following the injury, the patient noticed redness, pain, and vision loss in his right eye and visited the Ogasawara Clinic. Initial examination by the resident GP revealed severely impaired vision in the right eye, with visual acuity of the right eye limited to hand motion (n.c.) and an intraocular pressure (IOP) of 29.0 mmHg, measured using a non-contact tonometer.

The GP conducted a detailed anterior segment examination using a slit-lamp microscope equipped with a Smart Eye Camera (SEC; Smart Eye Camera ophthalmic examination device SLM-i08/SE2. Approval No. 13B2X10198030201, OUI Inc., Tokyo, Japan), capable of remote ophthalmic examinations.

The findings of the right eye included the following: eyelid swelling and redness; deep anterior chamber with hyphema and cells; conjunctival hyperemia; and normal corneal and lens appearance with no trauma-induced lens displacement. There are no significant findings of globe rupture through the cornea to the sclera. The anterior segment findings of the left eye were within normal limits. 

**Figure 1:**
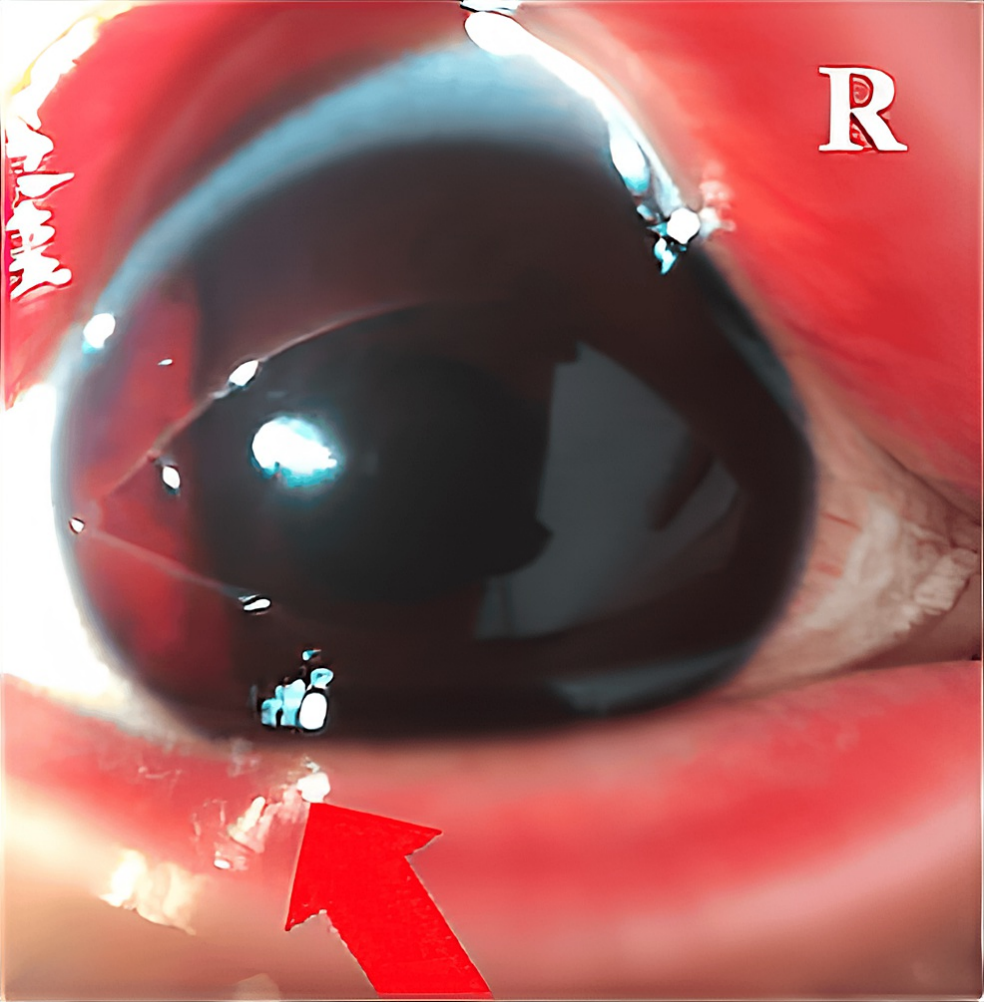
Teleophthalmology consultation from the resident general practitioner to an ophthalmologist in the mainland. An anterior segment video of the eyes. Eyelid swelling and redness, a deep anterior chamber containing hyphema and cells, conjunctival hyperemia, and a normal corneal and lens appearance with no sign of any trauma-induced lens.

Images and clinical data were transmitted to a mainland ophthalmologist through a telemedicine system for remote consultation. Fundus photography and orbital computed tomography (CT) imaging were also performed, revealing no translucent fundus or obvious orbital floor fractures. Due to the telemedicine consultation, the patient was advised to visit a mainland ophthalmologist (Figure 1).

**Figure 2:**
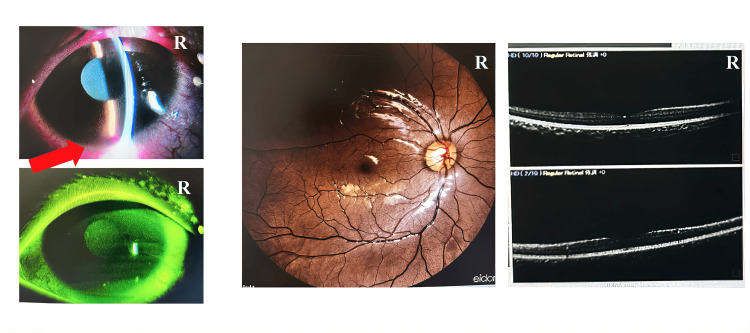
Findings in the ophthalmology facility. The anterior segment examination showed persistent but improving eyelid swelling and redness, along with reduced hyphema. Fundus examination revealed retinal commotio in the upper periphery without any signs of retinal detachment or optic nerve head swelling.

The next day, the patient traveled to the mainland. Upon arrival, a comprehensive ophthalmic examination was conducted (Figure 2).

The right eye's visual acuity had improved to 20/30 (n.c.). The IOP had decreased to 17.0 mmHg. The anterior segment examination showed persistent but improving eyelid swelling and redness, with reduced hyphema. There is no sign of any globe rupture through the cornea to the sclera.

Fundus examination revealed, retinal commotion in the upper periphery, but no retinal detachment or optic nerve head swelling as well as globe rupture. The OCT findings are normal. The findings in the left eye were within normal limits from the anterior segment to the retina and optic nerve.

Six days post injury, the patient was re-evaluated at the island clinic. His right eye visual acuity had further improved to 20/20 (n.c.), and IOP stabilized at 19.0 mmHg. The hyphema had resolved, and no abnormalities were noted in the fundus or optic nerve.

## Discussion

This case highlights the crucial role of telemedicine in managing emergencies in remote areas. Teleophthalmology enabled timely expert consultation and appropriate triage, facilitating the patient's transfer for specialized care [1,11]. The case underscores the potential of telemedicine to bridge the gap in healthcare accessibility in isolated regions, allowing for early intervention and improved patient outcomes. The integration of teleophthalmology in remote island settings such as Chichijima Island can significantly enhance the delivery of specialized care [12]. It provides an invaluable tool for resident GPs to access expert opinions and make informed decisions regarding patient management because of the lower incidence of visits to secondary care facilities in the Japanese isolated islands [13]​​​​​​​. This model of care is essential for regions with limited access to ophthalmologists, reducing the burden on patients to travel long distances for specialized care [12]​​​​​​​​​​​​​​.

In this case, a child with a contusion of the right eyeball due to a badminton shuttlecock was successfully managed through telemedicine. Ophthalmic trauma of this nature requires multiple examinations, including slit-lamp microscopy, fundus examination to evaluate retinal detachment and peripapillary optic nerve, central flicker test, pupillary response, CT scan, eye movement examination, etc. [14]​​​. Traumatic optic neuropathy and permanent visual dysfunction due to macular damage can also occur, necessitating close ophthalmologic examination and treatment. In this instance, the island clinic's capabilities included a slit-lamp microscope and CT examination, but the evaluation of the fundus and optic nerve required mainland consultation [15]​​. The recommendation for the patient to visit a mainland ophthalmologist was based on the following findings: Using the telemedicine system, the ophthalmologists were able to thoroughly examine the anterior segment of the eyes. Additionally, CT scans confirmed that there were no orbital bone fractures, ruling out one potential source of serious damage. However, despite the capabilities of the telemedicine system, it was not possible to replace fundus examination. Therefore, due to the inability to perform a complete fundus examination remotely, it was deemed essential for the patient to visit a mainland ophthalmologist for more detailed examinations.

Telemedicine, especially in ophthalmology, offers a practical solution to the lack of permanent ophthalmologists on many Japanese islands [2]​​​​​​​​​​​​​​. This invention significantly improves access to specialized services for remote populations, reduces the need for patient transfers, and saves on travel costs [16]​​​​​​​​​​​​​​. Tools such as the SEC facilitate remote diagnosis and have been effectively used in various settings [8]​​​​​​​​​​​​​. Functions of SEC have been demonstrated equally to the conventional slit-lamp microscopes in Japan [4-7]​​​​​​​​​​​​​​, Indonesia [8]​​​​​​​​​​​​​​, India [9]​​​​​​​​​​​​​, and Italy [10]​​​​​​​​​​​​​​. Therefore, it is used to diagnose anterior eye diseases such as corneal ulcers and provide care for severe dry eye cases, demonstrating its utility beyond remote islands outside of Japan [4-10]​​​​​​​​​​​​​​.

The development of portable ophthalmic medical equipment, especially smartphone-utilized devices, is crucial not only for remote islands but also for areas with a shortage of ophthalmic specialists [17]​​​​​​​​​​​​​​. The future of telemedicine will likely include the integration of artificial intelligence (AI) for diagnostic assistance, leveraging big data to enhance specialized treatment in remote and underserved areas [18-20]​​​​​​​​​​​​​​.

The successful management of this case through teleophthalmology underscores the transformative potential of telemedicine in providing high-quality care to patients in remote locations. Continued advancements in telemedicine technology and systems are essential to meet the growing healthcare needs of isolated populations.

## Conclusions

The integration of telemedicine allowed for the accurate diagnosis and effective management of traumatic hyphema, facilitating the patient's transfer to the mainland for further examination and treatment. The case underscores the necessity of developing and implementing robust telemedicine systems to bridge the gap in healthcare accessibility, particularly in regions with limited access to specialized medical care.

As medical technology continues to advance, the role of teleophthalmology will become increasingly vital in providing high-quality, timely care to patients in remote locations. The continued development of portable ophthalmic medical equipment and the incorporation of artificial intelligence for diagnostic support will further enhance the efficacy and reach of telemedicine. This case emphasizes the critical need for healthcare systems to adapt and expand their telemedicine capabilities to improve health outcomes for patients in remote and underserved communities.
